# Strategies for the functionalisation of gold nanorods to reduce toxicity and aid clinical translation

**DOI:** 10.7150/ntno.56432

**Published:** 2021-01-15

**Authors:** Xin Shi, Hannah L. Perry, James D. E. T. Wilton-Ely

**Affiliations:** Department of Chemistry, Imperial College London, Molecular Sciences Research Hub, White City Campus, London W12 0BZ, United Kingdom.

**Keywords:** Gold nanorods, functionalisation, cetrimonium bromide (CTAB), photothermal therapy

## Abstract

Gold nanorods (GNRs) show great promise as photothermal therapy agents due to their remarkable ability to convert light into heat. In most cases, gold nanorods are synthesised via a seed-mediated method assisted by surfactants. However, the toxicity of these surfactants, principally cetrimonium ions, has prevented GNRs from being used more widely *in vivo*. To address this issue, various detoxification and functionalisation approaches have been proposed in recent years to replace or cover surfactant coatings on the gold surface. In this short review, the advantages and limitations of each approach are examined in the context of the recent progress made towards the design of GNRs suitable for use in the body.

## 1. Introduction

Gold nanorods (GNRs) have attracted much attention due to their unique (photo)physical properties, which enable them to be utilised in a variety of applications such as photothermal therapy (PTT), biosensing and optical imaging 1-3. Among these applications, PTT is currently of greatest interest as it has the potential to offer a minimally-invasive therapeutic alternative to current cancer treatments, whereby tumour cells are killed by localised hyperthermia 4. Other hyperthermia methods include magnetic hyperthermia, radio-frequency hyperthermia, microwave hyperthermia and ultrasound hyperthermia, however, PTT shows particular promise due to the precision of this technique, which minimises damage to surrounding healthy tissue 5.

Gold nanorods show promise as PTT agents due to their ability to absorb light and transform the energy into heat, causing irreversible cellular damage and subsequent tumour regression 6. This ability to absorb light stems from the surface plasmon resonance (SPR) absorption of GNRs, which is the result of the collective resonance of the free electrons at the gold surface under incident radiation. The absorbed light is then converted into heat by electron-electron and electron-photon interactions 7; a process that boasts a conversion efficiency of up to 90% 8 and can result in localised temperatures as high as 77 °C 9. Crucially, for their application *in vivo*, the absorption wavelength of gold nanorods is within the near-infrared (NIR) region of the electromagnetic spectrum, which avoids the 'tissue window' in which endogenous chromophores absorb light.

Currently, there are several nanomaterial-based PTT agents that have shown promise in the treatment of cancer. Although most are in the early stages of development, a core-shell nanomaterial, known as AuroShell^®^ (Nanospectra Biosciences, TX, USA), has advanced to clinical trials 10. AuroShell^®^ nanoparticles feature a silica core 120 nm in diameter coated with a 12-15 nm layer of gold, which absorbs light in the NIR region of the electromagnetic spectrum. This approach relies on the enhanced permeability and retention (EPR) effect for accumulation of the AuroShell^®^ nanoparticles within the tumour following intravenous administration and has, so far, been used in the treatment of cancers of the head, neck, lung, breast and prostate 11,12. A second noteworthy gold nanomaterial, Aurimune-TNF (CytImmune Sciences, Rockville, MD), has also reached clinical trials as an anti-cancer agent 13. This 23 nm diameter gold nanomaterial is conjugated with human tumour necrosis factor α (TNF-α) via a polyethylene glycol linker and is designed to be ellipsoidal in shape, to red-shift its absorption wavelength towards the NIR region of the electromagnetic spectrum. Although not explicitly a PTT agent, a recent study reported an amplification of the biochemical action of Aurimune-TNF through a laser-induced photothermal effect in a target tumour 14. Some gold nanorod designs have been adapted to include imaging units on the nanorod surface. This facilitates the real-time tracking of the nanorods in the body and therefore ensures that the laser is applied only after substantial uptake of the nanorods, thereby maximising the efficacy of the PTT therapy 15.

In spite of their potent and efficient photothermal properties, gold nanorod-based PTT agents have yet to enter clinical trials. A significant contributor to this lack of clinical translation is the complex surface chemistry involved in maintaining the low toxicity of the nanorods whilst also promoting their stability towards aggregation. Although there are a number of straightforward routes to synthesising gold nanorods 1,16-18, the most popular approach is seed-mediated synthesis, which requires the use of chloroauric acid, cetrimonium bromide (CTAB), silver nitrate (to aid aspect ratio control), sodium borohydride and ascorbic acid. According to the proposed mechanism, CTAB, a cytotoxic surfactant that acts as a shape inducing agent and stabiliser, hinders the growth of the seed crystal on one axis and promotes growth on the orthogonal axis, producing a rod shape 19,20. Additionally, CTAB forms a bilayer structure around the GNRs, which increases their stability in aqueous solution.

**Figure 1:**
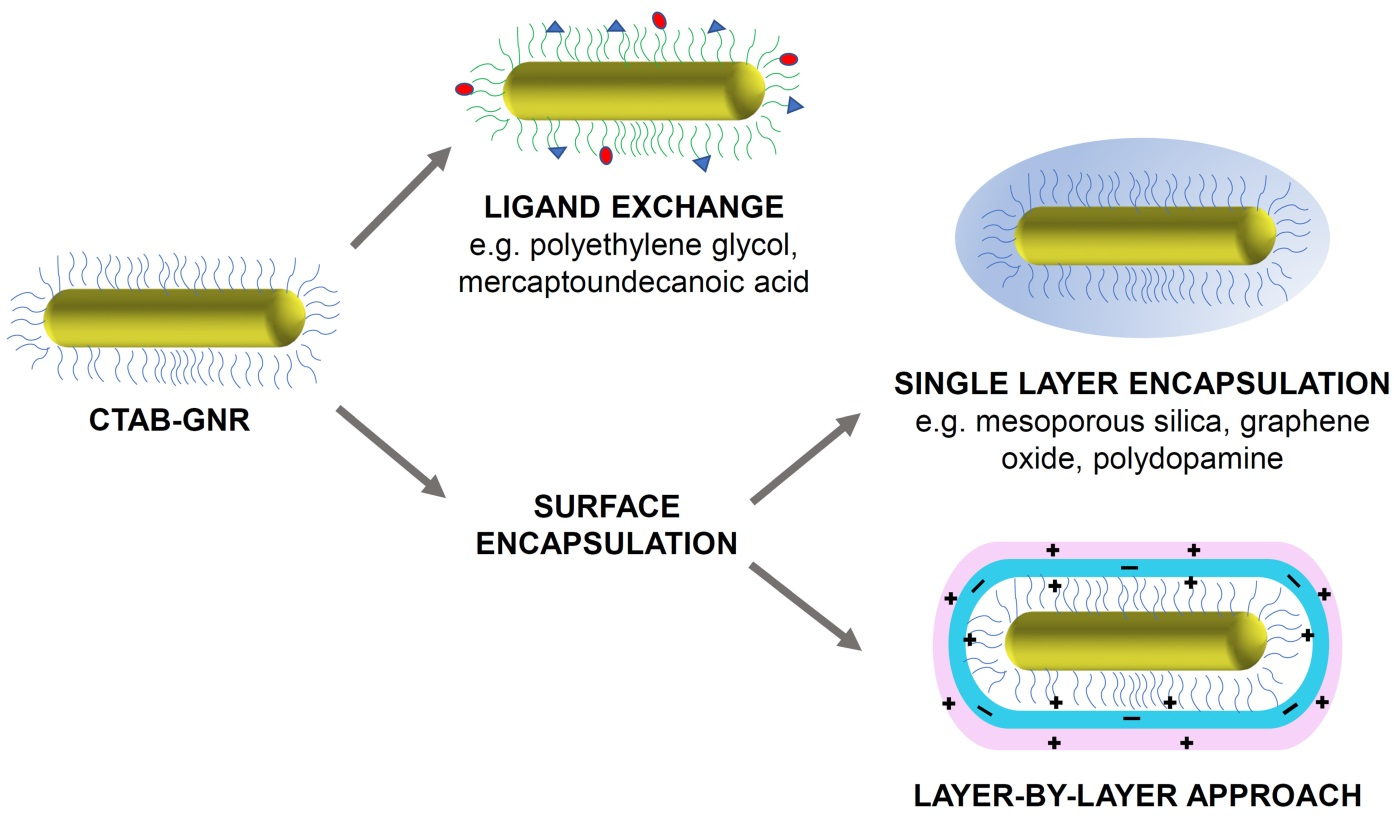
Illustration of some common strategies for the functionalisation and detoxification of CTAB-GNRs (original figure).

Despite the advantages of CTAB, its high cytotoxicity is a major concern and risks undermining any potential use of GNRs in a biological setting. The origin of this toxicity can be traced to the interactions of CTAB with the phospholipid bilayer of the cell and the inhibition of the enzyme ATP synthase by the cetrimonium cation, both of which lead to cell death 21. Therefore, for use in any biological application, the CTAB layer must either be exchanged with, or completely encapsulated by, a more biocompatible coating (Figure 1). This short review explores the progress made in the detoxification of CTAB-coated GNRs and aims to encourage further work in this area that may aid the translation of these highly promising PTT agents into a clinical setting. Many detoxification methods have been proposed and this review does not aim to replace previous reviews on the design of functionalised gold nanorods or claim to be an exhaustive survey of the field. Instead it focuses mainly on the approaches that can be used to mitigate the undesirable impact of CTAB.

## 2. Ligand exchange

Ligand exchange is one of the most widely used methods for removing CTAB from the surface of GNRs and involves replacing CTAB with organic ligands or biomolecules. The presence of these units increases the colloidal stability of the GNRs and protects them from aggregation, whilst also ensuring biocompatibility. As these ligands are designed to bind directly to the gold surface, rather than to coat the CTAB layer, they are designed to include thiol(ate) units due to the strong affinity of gold for soft, polarisable, sulfur donors.

### 2.1 Polyethylene glycol (PEG)

Among the many available surface ligands, polyethylene glycol thiols (PEG-SH), or the deprotonated form (thiolates), are arguably the most popular choice due to their many favourable properties and low cost. In addition to their hydrophilic nature, which enhances the suspension stability of the GNRs, PEG is also known to protect the GNRs from uptake by the immune system, which leads to a longer plasma circulation time, allowing the nanostructures greater opportunity to function as desired within the body. In addition, the end group (X) of X-PEG-SH can easily be linked to units providing further useful functions, such as targeting (e.g. antibodies, aptamers, peptides and small biomolecules) and therapy (e.g. chemotherapy drugs and monoclonal antibodies).

**Figure 2:**
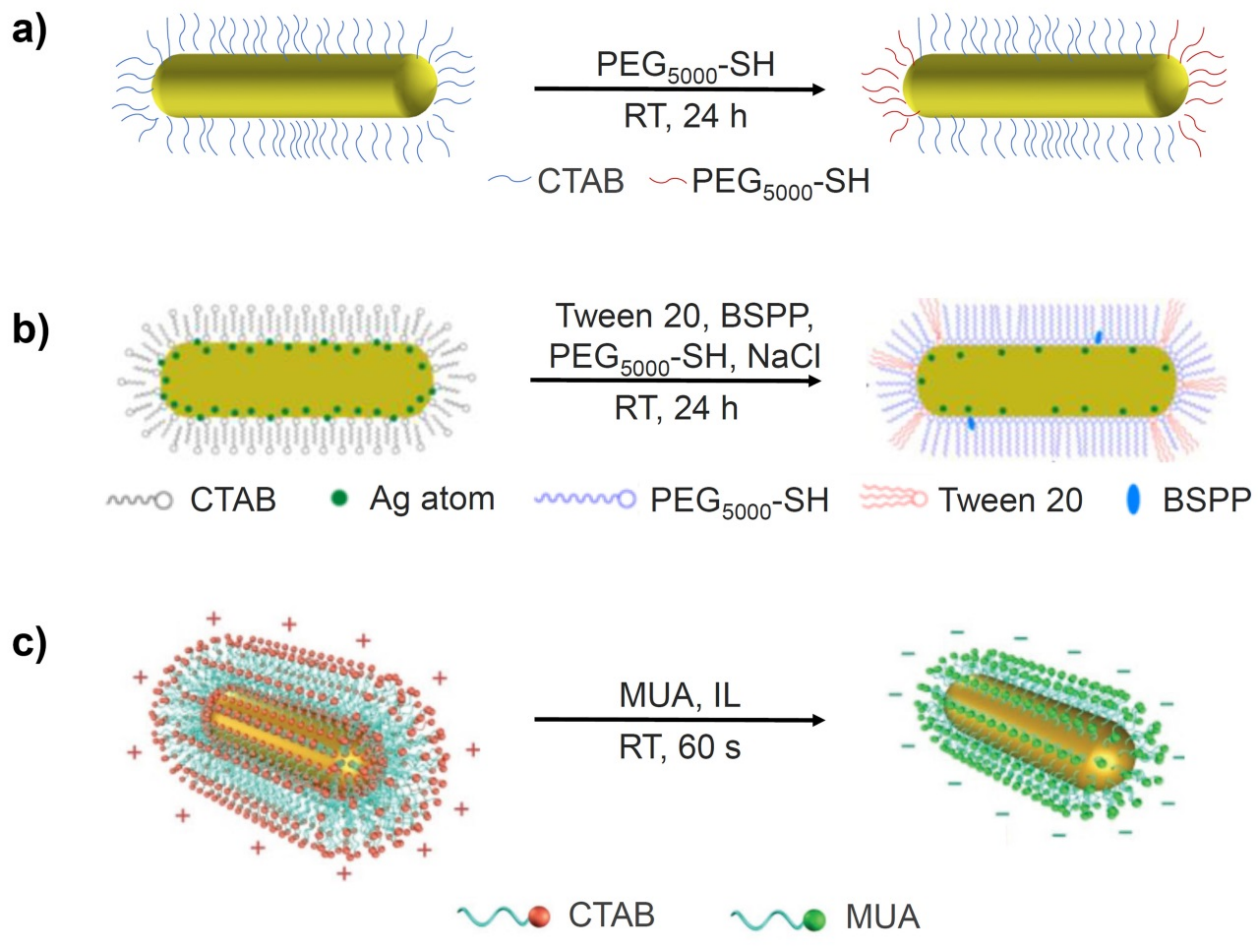
Schematic representation of (a) partially PEG-functionalised GNRs, (b) one-step surface functionalisation of GNRs with Tween 20, BSPP, PEG-SH and NaCl and (c) the preparation of MUA-GNR using an IL via the phase transfer method. Images adapted with permission from reference 27, copyright 2015 American Chemical Society and from reference 34, copyright 2017 John Wiley & Sons.

Among the many different ligand exchange methods proposed to replace CTAB with PEG-SH, the simplest is the one-step method, which involves addition of PEG-SH to a CTAB-GNR solution and stirring for 24 hours at room temperature (Figure 2a) 22. The major issue with this method, however, is that the resulting functionalised GNRs are less stable than their CTAB-GNR precursor and are therefore prone to aggregation. This method has been modified by adding Tris-HCl buffer (2-amino-2-hydroxymethyl-propane-1,3-diol, pH 3) to the reaction mixture to drive the loading process. The functionalised GNRs in this case were prepared by mixing CTAB-GNRs and PEG_5000_-SH in Tris-HCl buffer (pH 3) and stirring at room temperature for 30 min 23. The Tris-HCl buffer accelerates the loading of PEG onto the gold surface in two main ways. Firstly, the Tris amine group is able to replace the CTAB bilayer due to its affinity for the gold surface, which facilitates the interaction with PEG_5000_-SH. Secondly, the negative charge on the PEG molecules can be neutralised by the low pH of the Tris-HCl buffer, reducing the repulsion between PEG molecules, enhancing their rate of binding to the surface. The use of the Tris-HCl buffer results in a significant acceleration of the rate of reaction however, only the more weakly bound CTAB molecules at the ends of the GNRs are displaced by PEG_5000_-SH, producing only partially functionalised GNRs 24.

In order to increase the degree of ligand exchange, a two-step method has also been developed. After following the ligand exchange method described above, a second step is performed, in which PEG_5000_-SH is dissolved in a mixture of ethanol and water (90% v/v ethanol) and mixed with the material produced in the first step under gentle stirring for a further 24 hours 25. This enables the remaining CTAB to be desorbed by the ethanol and replaced with PEG_5000_-SH while maintaining the stability of the GNRs. The GNRs produced via this two-step method exhibit significantly lower *in vitro* toxicity compared to GNRs made via the one-step method and display a lower pro-inflammatory cytokine tumour necrosis factor (TNF-α), indicating a reduced inflammatory response.

Polysorbate 20 (commonly known as Tween 20) is a non-ionic surfactant, which has proved useful in the stabilisation of GNRs during the displacement of CTAB from their surfaces 26. It is used in conjunction with bis(*p*-sulfonatophenyl)phenylphosphine (BSPP), a surface activation agent that acts by exchanging with the CTAB bilayer before being replaced by PEG_2000_-SH and sodium chloride, which etches the silver pre-deposited on the GNR surface (Figure 2b), reducing the amount of residual silver. This powerful cocktail of reagents provides optimal conditions for the exchange of CTAB with PEG_2000_-SH in a one-step reaction. The result is complete functionalisation of the GNRs with PEG_2000_-SH and removal of most of the active silver on the GNR surface. Although it is difficult to quantify how much more CTAB is exchanged with PEG when using the Tween 20 method compared to other methods, cell viability assays have demonstrated that GNRs synthesised via the Tween 20 method exhibited far lower toxicity *in vitro* than GNRs synthesised using other methods, which suggests that these GNRs had a reduced CTAB content. For example, even at GNR concentrations as high as 80 μg/mL, their cytotoxicity was found to be approximately four times lower than the partially modified GNRs synthesised without Tween 20, BSPP and sodium chloride 27.

Whilst PEGylated nanomaterials show much promise, it is worth noting that they do not always achieve the biocompatibility necessary for progression towards clinical use 28-30. There is evidence that PEG itself may be immunogenic and induce the production of anti-PEG antibodies. These antibodies cause an immune response and accelerated blood clearance of the PEGylated nanomaterials, which reduces their circulation time and thus, in most cases, limits their efficacy 31. This is not the case for all PEGylated nanomaterials, however, as Doxil^®^, a PEGylated liposome loaded with doxorubicin, is non-immunogenic and was the first FDA-approved nano-drug 32.

### 2.2 Mercaptoundecanoic acid (MUA)

The linear molecule, 11-mercaptoundecanoic acid (MUA) is commonly used to stabilise gold nanomaterials. Attachment to the gold surface is achieved through a thiol(ate) group while the carboxylic acid end group enables biomolecules to be conjugated to the MUA layer in order to provide additional functionality. Coating with MUA confers biocompatibility on the GNRs without compromising the shape and size of the nanostructure.

Many protocols have been developed to functionalise GNRs with MUA with the most widely used being the 'round-trip' ligand exchange method. This involves transferring the GNRs into a different organic phase using solvents such as acetone, toluene and methanol. In a typical exchange process, dodecanethiol (DDT) is added to an aqueous CTAB-GNR solution to replace CTAB before adding acetone. The organic phase is then added to a solution of toluene and methanol (1:5) before centrifugation to remove excess DDT and CTAB. The DDT-GNRs are then re-suspended in toluene and mixed with MUA under reflux at 70 °C for 15 min. Finally, the MUA-coated GNRs are obtained after washing thoroughly with toluene and isopropanol 33.

More recently, a faster, more efficient version of this phase transfer method has been proposed, which uses a water-immiscible ionic liquid (IL), 1-butyl-3-methylimidazolium bis(trifluoromethylsulfonyl)imide ([bmim][NTf_2_]), to achieve ligand exchange in as little as 2 minutes (Figure 2c). Briefly, GNRs are first transferred from the aqueous phase to the IL layer by vigorous shaking, aided by the polarity of the IL. A solution of MUA in the IL is then added, resulting in a reverse phase transfer whereby MUA-GNR forms in the aqueous layer due to the hydrophilicity of the carboxylic acid groups now present on the GNR surface. A major benefit of this approach is that, unlike with PEG-GNRs, MUA-GNRs can be obtained in large quantities and stored as powders for later use. However, most importantly, GNRs functionalised with MUA are reported to maintain good colloidal stability 34.

A more environmentally friendly approach has been proposed that avoids the use of organic solvents. The functionalisation was achieved by mixing the CTAB-GNR material with MUA in a pH 10 solution overnight. The high basicity of the solution used in this case is advantageous as it maintains the colloidal stability of both the starting material (CTAB-GNR) and the final product (MUA-GNR) 35. Although it is desirable to limit the use of organic solvents in synthesis, this protocol is reportedly difficult to control and can lead to irreversible aggregation due to destabilisation of the CTAB bilayer before addition of the new surface ligand.

### 2.3 Alternative ligands

Although PEG and MUA are arguably the most commonly used ligands employed in the exchange of CTAB on the surface of GNRs, alternatives, such as thiolated polyamidoamine (PAMAM) 36 and disulfide polysarcosine (PS) 37 have also been explored. PAMAM is a dendrimer capable of completely replacing surface-bound CTAB to produce nanorods that display greater dispersion in water than their CTAB-GNR precursor 36. Polysarcosine (PS) is a nonionic, hydrophilic polypeptoid, consisting of repeating units of the natural amino acid, sarcosine (also known as *N*-methylglycine). The use of this surface unit is an exciting prospect in the functionalisation of GNRs as it has been reported that the resultant materials exhibit extended blood circulation times compared to PEG-GNRs, resulting in greater tumour uptake and thus a greater PTT effect 37. The widespread use of PEG for GNR ligand exchange is likely to continue due to its low cost and the established exchange protocols. However, with the appearance of further publications reporting high aqueous stability and greater tumour uptake for the alternatives could make their use more commonplace.

## 3. Encapsulation methods

Improved biocompatibility has also been achieved by the encapsulation of CTAB-GNRs with inorganic or carbon-based materials to form 'nanoshells'. Nanoshells of this nature are typically made of mesoporous silica, graphene or carbon nanotubes. These surface coatings enhance the chemical stability of CTAB-GNRs whilst providing a potentially more robust alternative to polymer-based coatings. Furthermore, as seen with the various coatings described above, nanoshells can be modified to incorporate surface ligands that can provide additional functionality for targeting and therapy.

### 3.1 Mesoporous silica

Mesoporous silica (mSiO_2_) is the most widely used encapsulation material due to the facile nature of the coating protocols and the large surface area available for loading with therapeutic drugs and other useful ligands 38,39. The mSiO_2_ coating is usually applied to GNRs by adding tetraethyl orthosilicate (TEOS) under basic conditions allowing the thickness of the coating to be controlled simply by reducing or extending the reaction time 40.

Crucially for application in photothermal therapy (PTT), the mesoporous silica shell appears not to inhibit the absorption of light and hyperthermia-induced cell death can be achieved with these coated nanomaterials. Moreover, one report has provided evidence that the mSiO_2_ coating actually enhances the PTT efficacy compared to uncoated GNRs, as the coating shifts the longitudinal SPR peak closer to the wavelength of the laser, resulting in a more intense SPR effect 40. The enhanced cellular uptake of mSiO_2_-GNRs relative to their PEG-coated analogues has also been identified as a factor in the enhanced PTT effect observed 41. Although these are encouraging results, it is worth also considering the effect of the mSiO_2_ layer on the transfer of heat to the surrounding tissue. Even if the mSiO_2_ coating increases absorption of light and uptake by cells, it will also increase the distance between the source of heat and the tissue, which could potentially reduce the actual temperature to which the tissue will be exposed, reducing the efficacy of the PTT. However, the *in vivo* PTT studies performed with mSiO_2_-GNRs show great promise and provide a solid foundation for future research 42.

Another consideration in the application of mSiO_2_ coatings is their poor degradability, which stems from the strength of the Si-O-Si framework. Hydroxyapatite (Ca_10_(PO_4_)_6_(OH)_2_, HAP) has been used to improve the biodegradability and pH-sensitive drug release properties of mSiO_2_-GNRs. Moreover, after application of a 20 nm thick HAP/mSiO_2_ shell, a zeta potential greater than that of mSiO_2_-GNR was observed due to the strongly electronegative HAP in the inorganic shell, which improved the colloidal stability of the GNRs. Characterisation of these materials reveals that parts of the Si-O-Si framework is replaced with Si-O-Ca units when the calcium salt is added to the mesoporous silica shells. At a tumour site, where the local pH environment is more acidic than in healthy tissues, HAP is easily eroded, resulting in the removal of Ca^2+^ ions from within the Si-O-Ca structure. The defects generated in this manner within the silica framework enable the controlled release of the chemotherapy drug, Doxorubicin (DOX), and facilitate the collapse of the silica shell and thus enhance the biodegradability of the mSiO_2_-GNRs 43.

**Figure 3:**
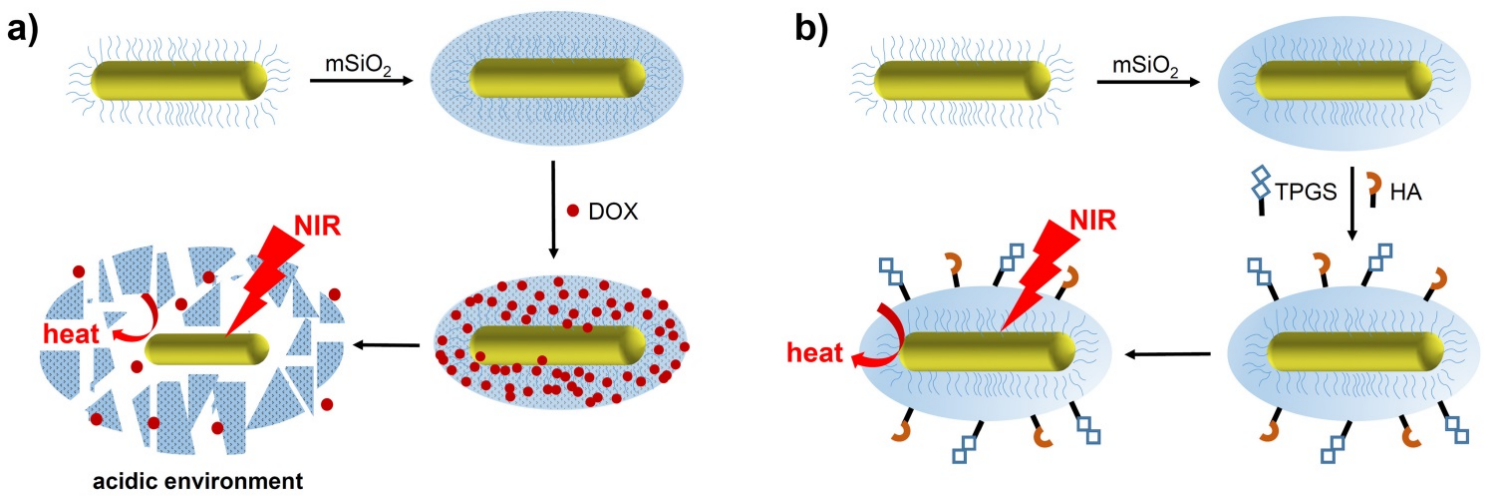
Preparation and therapeutic activation of (a) DOX-mSiO_2_-GNRs and (b) TPGS-HA-mSiO_2_-GNRs (original image).

The potential for drug release has been explored using mSiO_2_-GNRs doped with Doxorubicin (DOX), which has been proposed as a particularly potent anti-cancer nanomedicine capable of combined chemo- and photothermal therapy (Figure 3a) 43, 44. The mSiO_2_ coating benefits from a high loading capacity, meaning that more DOX per nanorod can be delivered to the target site, resulting in a greater chemotherapeutic effect. Additionally, targeting moieties such as hyaluronic acid (HA) and D-α-tocopherol polyethylene glycol 1000 succinate (TPGS) have been employed to target mSiO_2_-GNRs to certain tumours (Figure 3b). After being coated with the mSiO_2_ shell, the width of the gold nanostructures increased to around 18 nm. This approach aims to increase nanorod concentration within the tumour and therefore increase the efficacy of the PTT 45.

### 3.2 Encapsulation with metal chalcogenides

Although mSiO_2_ is the most widely used inorganic material for CTAB-GNR encapsulation, other coatings have been explored which often provide their own distinct benefits. For example, it has been reported that the addition of a silver layer between the CTAB-GNRs and the SiO_2_ layer facilitates a responsive amplification of photoacoustic signal when in the presence of prostate specific antigen. This strategy was tested using an enzyme-linked immunosorbent assay (ELISA) system for detecting prostate specific antigen (PSA). Once the PSA antibody was detected, the hydrogen peroxide generated from the ELISA system etches the silver layer in the nanostructure, thus producing silver ions, leading to an enhanced photoacoustic signal at 780 nm 46. Manganese(IV) oxide, MnO_2_ 47, has also been of interest due to its ability to release small amounts of paramagnetic Mn^2+^ ions, which can be used to enhance contrast in magnetic resonance imaging (MRI). However, elevated levels of Mn^2+^ can cause a toxic effect in humans (manganism) so a greater understanding of the form (e.g., chelated or unchelated) in which these ions are released would be needed before this approach can be translated to the clinic. Encapsulation by a copper sulfide (Cu_7_S_4_) has been reported to result in an enhancement in photothermal conversion efficiency and photothermal stability compared to CTAB-GNRs, however synthesis of this nanomaterial is challenging 48. In contrast, copper(II) oxide-coated CTAB-GNRs have been synthesised via a more straightforward route and the electrochemical attributes of this coating have been utilised to sense glucose in solution 49. CTAB-GNRs coated with ZnO have shown early promise as a particularly potent anti-cancer agent, as the coating facilitates the production of near-infrared light-induced singlet oxygen, affording this nanomaterial the ability to treat cancer simultaneously by both PTT and photodynamic therapy (PDT) 50.

The introduction of additional metallic elements or metal oxides and sulfides into the design is also a potential cause for concern in terms of toxicity. However, the unique benefits that these coatings are able to provide warrant their continued investigation if the toxicity aspect is monitored. Whilst mSiO_2_ remains the most widely-used coating material for the encapsulation of CTAB-GNRs, the emergence of innovative alternatives provides further options for functional coatings. It must also be recognised that the additional complexity of these systems could also present a significant drawback to their widespread use by researchers outside the field of materials science.

### 3.3 Graphene oxide and derivatives

Graphene oxide (GO) is an alternative coating material for GNRs, which facilitates imaging through surface enhanced Raman spectroscopy (SERS) whilst also offering high stability and biocompatibility. The adsorption of GO onto the surface of GNRs occurs through interaction of the negative charge on the GO sheet with the positively charged GNRs 51. A typical synthesis involves the addition of a GO solution to a CTAB-GNR dispersion, followed by continuous stirring at room temperature.

**Figure 4:**
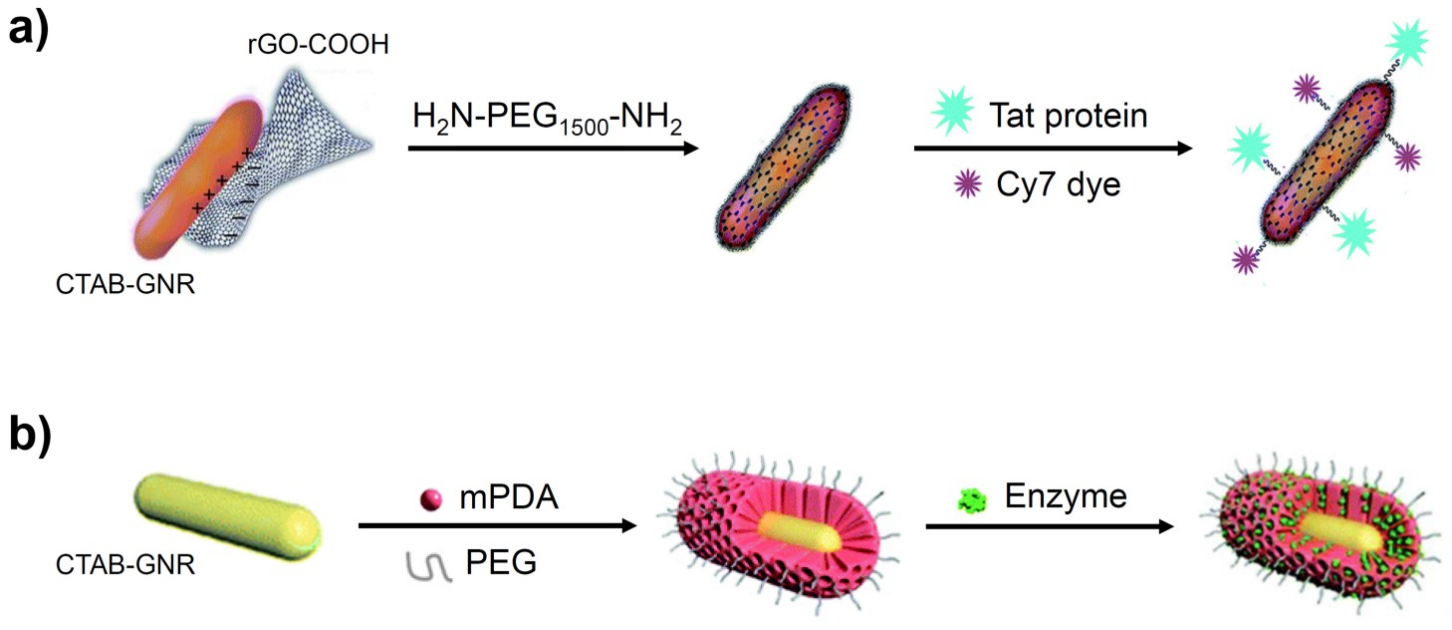
Depiction of CTAB-GNRs encapsulated with (a) graphene oxide and (b) polydopamine. Images adapted with permission from 53, copyright 2016 Royal Society of Chemistry and from 57, copyright 2020 Royal Society of Chemistry.

GO-GNRs have been employed as vehicles for many anticancer agents, due to the strong adsorption of aromatic molecules onto the graphene sheets. Various NIR probes have also been linked to the GO surface to generate enhanced NIR SERS activity 52. Reduced GO, which has a lower oxygen content, provides an alternative option to cover the toxic CTAB layer. As demonstrated in Figure 4a, for example, GNRs have been coated with reduced GO, without any significant change in their size, and further modified with H_2_N-PEG_1500_-NH_2_, to increase the dispersibility, the Tat protein, for tumour-targeting, and a cyanine dye (Cy7), for fluorescence imaging 53.

The graphene oxide coating method, as with all other coating methods, involves simply covering the CTAB layer, rather than replacing it, which inevitably attracts toxicity concerns due to the continued presence of cetrimonium ions within the assembly. Furthermore, it has been reported that graphene and graphene-related materials can have an undesirable effect on various biological mechanisms 54,55. Further investigation is required into these effects (especially longer-term) before graphene oxide coatings are likely to be embraced more widely.

### 3.4 Polydopamine

Polydopamine (PDA), often in combination with PEG, has been used in both ligand exchange and surface encapsulation protocols, owing to its facile shell formation, high stability and unique molecular adsorption properties 56,57. Figure 4b shows CTAB-GNRs encapsulated by mesoporous polydopamine (mPDA) and PEG, resulting in coated GNRs with a shell thickness of around 30 nm. This nanomaterial was found to show enhanced stability towards aggregation (UV-vis and zeta potential measurements) and reached a higher temperature on irradiation with 1064 nm light than simple mPDA-coated GNRs. An enzyme was also incorporated into the design to assist in the degradation of the tumour, resulting in a greater uptake of the GNRs and hence a greater efficacy of PTT 57.

### 3.5 Albumin

Some proteins, particularly human serum albumin (HSA) 58 and bovine serum albumin (BSA) 59, have been employed in the encapsulation of GNRs. HSA and BSA have been used extensively to coat a variety of nanomaterials due to their biocompatibility, water compatibility and degradability. However, albumin is vulnerable to temperature rises, which can induce changes in the conformation of the protein, resulting in the exposure of CTAB, leading to associated toxicity issues. Thermal aggregation of BSA has been studied and it was revealed that this conformational change occurs at around 60 °C 60-62. Given that PTT can cause the generation of localised temperatures well above this value, albumin may not be the most suitable coating for GNR designs for use in PTT. However, BSA and HSA could still serve a useful role in nanomaterials where no heating effect is anticipated.

## 4. Multiple charged layers

The layer-by-layer (LbL) approach has been demonstrated to be a versatile surface modification strategy whereby the entire positively charged CTAB-GNR surface is coated by multiple, alternating, charged layers. This simple method for fabricating multilayers not only effectively encapsulates the toxic CTAB but also allows the incorporation of drugs, genes and proteins into the same assembly through electrostatic interactions.

**Figure 5:**
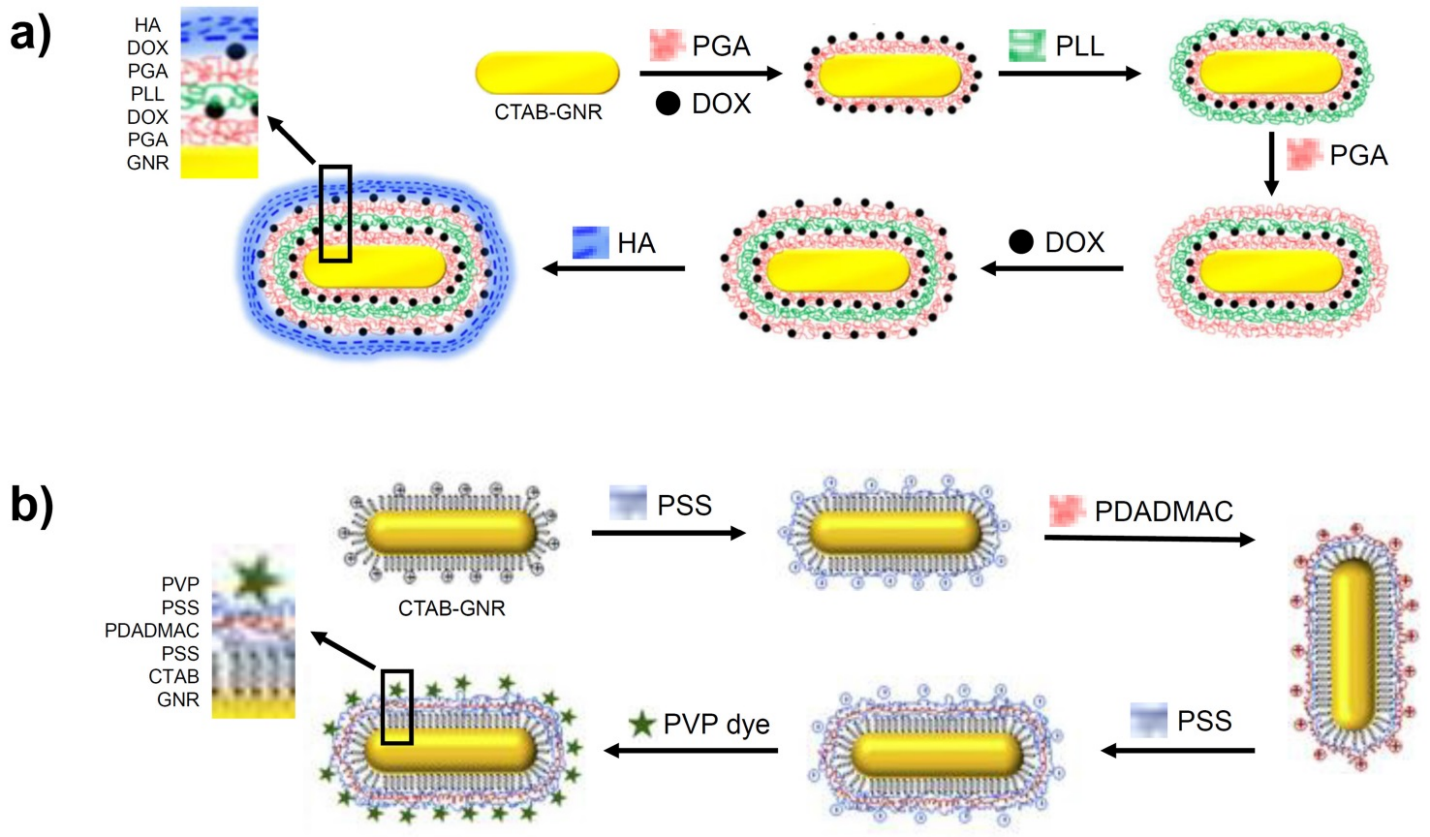
Formation of GNRs coated in multiple layers using the LbL method. Images adapted from reference 63, with permission from Portland Press, and from reference 64, with permission from Elsevier.

A targeted, multi-layered nanostructure has been prepared using the LbL strategy that combines the delivery of the chemotherapeutic, Doxorubicin (DOX) with GNR-based PTT 63. As shown in Figure 5a, the CTAB-GNR solution is incubated with a solution of negatively charged poly-glutamic acid (PGA), leading to a monolayer and the reversal of the surface charge. This is followed by the adsorption of the cationic DOX unit onto the PGA layer, followed by addition of poly-L-Lysine (PLL), resulting in a positively charged nanostructure. Coating with PGA and DOX is repeated before finally applying a hyaluronic acid (HA) layer for targeting the overexpressed CD44 receptors in ovarian cancer cells. After the multi-layer functionalisation, the nanomaterial increased from 40 nm in length for the CTAB-GNRs to an overall diameter of 80 nm for the LbL functionalised nanostructure. On irradiation of the multi-layered nanorod with NIR light, release of DOX is observed from the assembled layers, which complements the PTT. An *in vitro* cytotoxicity assay performed on SKOV3 ovarian cancer cells revealed the increased potency of the coated GNRs (IC_50_ of 0.35 μg/mL) compared to free DOX (IC_50_ of 1.35 μg/mL) and the coated GNRs in the absence of NIR irradiation (IC_50_ of 1.31 μg/mL) 63. These results illustrate the benefit of treating cancer using a combined chemo- and photothermal approach as well as the role of nanomaterials in delivering this therapy.

A further example of a multi-layered GNR synthesised via the LbL method features layers of poly(styrene) sulfonate (PSS) and poly(diallyldimethylammonium chloride) (PDADMAC) onto which a (phenothiazinyl)vinyl-pyridinium (PVP) chromophore is adsorbed (Figure 5b). These three polyelectrolyte layers had less of an effect on the nanorod size compared to the approaches described above, adding just 7-8 nm to the size of the GNR. The addition of this chromophore to the GNR generates a theranostic nanomaterial capable of both optical imaging and PTT 64.

Although the LbL method provides a relatively straightforward route for the preparation of stable gold nanorods, the fact that CTAB remains on the nanorod surface is a cause for concern. It is believed that the encapsulation of CTAB is sufficient to nullify its toxic effect, however, this is yet to be confirmed in detailed studies. Thorough evaluation of the effect of residual CTAB on the toxicity of these multi-layered nanorods is required to enable their progression towards clinical use.

Table 1 brings together the various approaches covered in this review to provide a guide to the advantages and disadvantages of the various strategies employed for the detoxification of CTAB-GNRs through surface functionalisation.

## Conclusion

The unique photophysical properties of GNRs make them highly efficient transducers of light energy into heat and thus promising candidates for use in photothermal therapy (PTT). Surfactants, such as CTAB, are necessary reagents in the synthesis of GNRs as they are required to direct the rod shape and stabilise the nanorods towards aggregation. However, the cytotoxicity of CTAB requires the careful replacement or covering of surfactant layers before use *in vivo* and this issue needs to be addressed thoroughly before clinical application can be considered.

This review summarises key examples drawn from the widespread efforts being made to functionalise and detoxify GNRs so that they may be used safely in humans as PTT agents. The most commonly used approach employs the displacement of CTAB with PEG, as this method is conceptually straightforward and the biocompatibility of the final product is acceptable. Although much progress has been made in this area, the complete replacement of surfactant units is often a significant challenge, necessitating additional steps to avoid only partial removal.

More recent approaches based on the encapsulation of CTAB under further layers have attracted significant attention. This approach also offers a wide range of options to attach further therapeutic, targeting or imaging functionality to the surface, which can assist in achieving more accurate and effective cancer treatments. Of these designs, mSiO_2_-coated GNRs are the most widely used due to the high loading of surface units that can be achieved. Another benefit of mSiO_2_ and other single-layer coatings is the simplicity of the final product compared to GNRs functionalised via a LbL approach, which improves the reproducibility of the system and makes clinical translation more straightforward. However, given that CTAB remains on the surface of these coated GNRs, further demonstration of their benefits, particularly in view of the additional synthetic steps, is necessary to assuage concerns over residual toxicity caused by the retention of the CTAB under the surface layers 1-5.

Much of the work described here has been limited to *in vitro* testing, with only a handful progressing to *in vivo* studies. Therefore, in order for GNRs to move closer to wider clinical translation, it is important to investigate their behaviour and function in a biological context. It is hoped that with more preclinical data, GNR materials will follow the nanomaterials, AuroShell^®^ and Aurimune-TNF, and progress to clinical trials in the near future.

## Figures and Tables

**Table 1:** Summary of the advantages and disadvantages of various strategies for the functionalisation and detoxification of CTAB-GNRs.

Functionalisation strategies		Advantages	Disadvantages	Ref.
**Ligand exchange**	Polyethylene glycol	Ease of further modification, hydrophilicity	Possible immunogenic response	21-30
	Mercaptoundecanoic acid	Colloidal stability	Lack of versatility	31-33
	Polyamidoamine	Ease of further modification	Toxicity risk	34
	Polysarcosine	Colloidal stability	Lack of versatility	35
**Single layer encapsulation**	mSiO_2_	High loading capability	Poor biodegradability	36,37,41
	CuO	Ease of further modification	Less versatile, toxicity risk	47
	MnO_2_, Cu_7_S_4_, ZnO	Additional specific functions	Less versatile, toxicity risk	45,46,48
	Graphene oxide	Colloidal stability	Safety concerns	51-53
	Polydopamine	Ease of further modification	Safety concerns	54,55
	Albumin	Biocompatibility, degradable	Lack of thermal stability	56-60
**Layer-by-layer approach**	PGA, PLL, PSS, PDADMAC	Colloidal stability, potential for high loadings	Difficult modification, lack of versatility	61,62

PGA = Poly-glutamic acid, PLL = poly-L-Lysine, PSS = poly(styrene) sulfonate, PDADMAC = poly(diallyldimethylammonium chloride).

## References

[1] Zhou J, Cao Z, Panwar N, Hu R, Wang X, Qu J (2017). Functionalized gold nanorods for nanomedicine: Past, present and future. Coord Chem Rev.

[2] Yang DP, Cui DX (2008). Advances and prospects of gold nanorods. Chem Asian J.

[3] An L, Wang Y, Tian Q, Yang S (2017). Small gold nanorods: recent advances in synthesis, biological imaging, and cancer therapy. Materials.

[4] Chen J, Ning C, Zhou Z, Yu P, Zhu Y, Tan G (2019). Nanomaterials as photothermal therapeutic agents. Prog Mater Sci.

[5] Beik J, Abed Z, Ghoreishi FS, Hosseini-Nami S, Mehrzadi S, Shakeri-Zadeh A (2016). Nanotechnology in hyperthermia cancer therapy: From fundamental principles to advanced applications. J Controlled Release.

[6] Guo D, Huang Y, Jin X, Zhang C, Zhu X (2020). A Redox-Responsive, *In-situ* polymerized polyplatinum (IV)-coated gold nanorod as an amplifier of tumor accumulation for enhanced thermo-chemotherapy. Biomaterials.

[7] Huang X, Neretina S, El-Sayed MA (2009). Gold nanorods: from synthesis and properties to biological and biomedical applications. Adv Mater.

[8] Ahmad R, Fu J, He N, Li S (2016). Advanced gold nanomaterials for photothermal therapy of cancer. J Nanosci Nanotechnol.

[9] Huang X, Jain PK, El-Sayed IH, El-Sayed MA (2006). Determination of the minimum temperature required for selective photothermal destruction of cancer cells with the use of immunotargeted gold nanoparticles. Photochem Photobiol.

[10] Rastinehad AR, Anastos H, Wajswol E, Winoker JS, Sfakianos JP, Doppalapudi SK (2019). Gold nanoshell-localized photothermal ablation of prostate tumors in a clinical pilot device study. PNAS.

[11] Kim E-M, Jeong H-J (2017). Current status and future direction of nanomedicine: focus on advanced biological and medical applications. Nucl Med Mol Imaging.

[12] Ali MR, Wu Y, El-Sayed MA (2019). Gold-nanoparticle-assisted plasmonic photothermal therapy advances toward clinical application. J Phys Chem C.

[13] Pillai G (2004). Nanomedicines for cancer therapy: an update of FDA approved and those under various stages of development. SOJ Pharm Pharm Sci.

[14] Shao J, Griffin RJ, Galanzha EI, Kim J-W, Koonce N, Webber J (2013). Photothermal nanodrugs: potential of TNF-gold nanospheres for cancer theranostics. Sci Rep.

[15] Perry HL, Botnar RM, Wilton-Ely JD (2020). Gold nanomaterials functionalised with gadolinium chelates and their application in multimodal imaging and therapy. Chem Commun.

[16] Lohse SE, Murphy CJ (2013). The quest for shape control: a history of gold nanorod synthesis. Chem Mater.

[17] Vigderman L, Khanal BP, Zubarev ER (2012). Functional gold nanorods: synthesis, self-assembly, and sensing applications. Adv Mater.

[18] Liu A, Wang G, Wang F, Zhang Y (2017). Gold nanostructures with near-infrared plasmonic resonance: Synthesis and surface functionalization. Coord Chem Rev.

[19] Murphy CJ, Sau TK, Gole AM, Orendorff CJ, Gao J, Gou L (2005). Anisotropic metal nanoparticles: synthesis, assembly, and optical applications. J Phys Chem B.

[20] Park K, Drummy LF, Wadams RC, Koerner H, Nepal D, Fabris L (2013). Growth mechanism of gold nanorods. Chem Mater.

[21] Ito E, Yip KW, Katz D, Fonseca SB, Hedley DW, Chow S (2009). Potential use of cetrimonium bromide as an apoptosis-promoting anticancer agent for head and neck cancer. Mol Pharmacol.

[22] Niidome T, Yamagata M, Okamoto Y, Akiyama Y, Takahashi H, Kawano T (2006). PEG-modified gold nanorods with a stealth character for *in vivo* applications. J Controlled Release.

[23] Zhang Z, Lin M (2014). Fast loading of PEG-SH on CTAB-protected gold nanorods. RSC Adv.

[24] Du X, Lin W-C, Shou Q, Liang X, Liu H (2019). pH optimization for high-efficiency PEGylation of gold nanorods. Colloid Polym Sci.

[25] Kinnear C, Dietsch H, Clift MJ, Endes C, Rothen-Rutishauser B, Petri-Fink A (2013). Gold nanorods: controlling their surface chemistry and complete detoxification by a two-step place exchange. Angew Chem Int Ed.

[26] Li J, Zhu B, Zhu Z, Zhang Y, Yao X, Tu S (2015). Simple and rapid functionalization of gold nanorods with oligonucleotides using an mPEG-SH/Tween 20-assisted approach. Langmuir.

[27] Liu K, Zheng Y, Lu X, Thai T, Lee NA, Bach U (2015). Biocompatible gold nanorods: One-step surface functionalization, highly colloidal stability, and low cytotoxicity. Langmuir.

[28] Sebak A (2018). Limitations of pegylated nanocarriers: unfavourable physicochemical properties, biodistribution patterns and cellular and subcellular fates. Int J Pharm.

[29] Zhang P, Sun F, Liu S, Jiang S (2016). Anti-PEG antibodies in the clinic: Current issues and beyond PEGylation. J Controlled Release.

[30] Kozma GT, Mészáros Ts, Vashegyi I, Fülöp Ts, Örfi E, Dézsi L (2019). Pseudo-anaphylaxis to polyethylene glycol (PEG)-coated liposomes: roles of anti-PEG IgM and complement activation in a porcine model of human infusion reactions. ACS Nano.

[31] Zhang F, Liu M-R, Wan H-T (2014). Discussion about several potential drawbacks of PEGylated therapeutic proteins. Biol Pharm Bull.

[32] Barenholz YC (2012). Doxil®—the first FDA-approved nano-drug: lessons learned. J Controlled Release.

[33] Li D, Zhang M, Xu F, Chen Y, Chen B, Chang Y (2018). Biomimetic albumin-modified gold nanorods for photothermo-chemotherapy and macrophage polarization modulation. Acta Pharm Sin B.

[34] Su L, Hu S, Zhang L, Wang Z, Gao W, Yuan J (2017). A fast and efficient replacement of CTAB with MUA on the surface of gold nanorods assisted by a water-immiscible ionic liquid. Small.

[35] Caño R, Gisbert-González JM, González-Rodríguez J, Sánchez-Obrero G, Madueño R, Blázquez M (2020). Effective replacement of cetyltrimethylammonium bromide (CTAB) by mercaptoalkanoic acids on gold nanorod (AuNR) surfaces in aqueous solutions. Nanoscale.

[36] Li Z, Huang P, Zhang X, Lin J, Yang S, Liu B (2010). RGD-conjugated dendrimer-modified gold nanorods for *in vivo* tumor targeting and photothermal therapy. Mol Pharm.

[37] Zhu H, Chen Y, Yan F-J, Chen J, Tao X-F, Ling J (2017). Polysarcosine brush stabilized gold nanorods for *in vivo* near-infrared photothermal tumor therapy. Acta Biomater.

[38] Huang P, Bao L, Zhang C, Lin J, Luo T, Yang D (2011). Folic acid-conjugated silica-modified gold nanorods for X-ray/CT imaging-guided dual-mode radiation and photo-thermal therapy. Biomaterials.

[39] Li C, Zhang Y, Li Z, Mei E, Lin J, Li F (2018). Light-responsive biodegradable nanorattles for cancer theranostics. Adv Mater.

[40] Huang Q, Zou Y, Zhong S, Yang X, Li J, Huang W (2019). Silica-coated gold nanorods with high photothermal efficiency and biocompatibility as a contrast agent for *in vitro* terahertz imaging. J Biomed Nanotechnol.

[41] Zhu X-M, Fang C, Jia H, Huang Y, Cheng CH, Ko C-H (2014). Cellular uptake behaviour, photothermal therapy performance, and cytotoxicity of gold nanorods with various coatings. Nanoscale.

[42] Fang S, Lin J, Li C, Huang P, Hou W, Zhang C (2017). Dual-stimuli responsive nanotheranostics for multimodal imaging guided trimodal synergistic therapy. Small.

[43] Song Z, Liu Y, Shi J, Ma T, Zhang Z, Ma H (2018). Hydroxyapatite/mesoporous silica coated gold nanorods with improved degradability as a multi-responsive drug delivery platform. Mater Sci Eng C Mater Biol Appl.

[44] Xu C, Chen F, Valdovinos HF, Jiang D, Goel S, Yu B (2018). Bacteria-like mesoporous silica-coated gold nanorods for positron emission tomography and photoacoustic imaging-guided chemo-photothermal combined therapy. Biomaterials.

[45] Jacinto TA, Rodrigues CF, Moreira AF, Miguel SP, Costa EC, Ferreira P (2020). Hyaluronic acid and vitamin E polyethylene glycol succinate functionalized gold-core silica shell nanorods for cancer targeted photothermal therapy. Colloids Surf B Biointerfaces.

[46] Jiang C, Huang Y, He T, Huang P, Lin J (2020). A dual-round signal amplification strategy for colorimetric/photoacoustic/fluorescence triple read-out detection of prostate specific antigen. Chem Commun.

[47] Wang L, Li D, Hao Y, Niu M, Hu Y, Zhao H (2017). Gold nanorod-based poly (lactic-co-glycolic acid) with manganese dioxide core-shell structured multifunctional nanoplatform for cancer theranostic applications. Int J Nanomedicine.

[48] Leng C, Zhang X, Xu F, Yuan Y, Pei H, Sun Z (2018). Engineering gold nanorod-copper sulfide heterostructures with enhanced photothermal conversion efficiency and photostability. Small.

[49] Tang Y, Liu Q, Yang X, Wei M, Zhang M (2017). Copper oxide coated gold nanorods like a film: a facile route to nanocomposites for electrochemical application. J Electroanal Chem.

[50] Zhou N, Zhu H, Li S, Yang J, Zhao T, Li Y (2018). Au nanorod/ZnO Core-Shell nanoparticles as nano-photosensitizers for near-infrared light-induced singlet oxygen generation. The J Phys Chem C.

[51] Shirshahi V, Hatamie S, Tabatabaei SN, Salimi M, Saber R (2018). Enhanced thermal stability and biocompatibility of gold nanorods by graphene oxide. Plasmonics.

[52] Qiu X, You X, Chen X, Chen H, Dhinakar A, Liu S (2017). Development of graphene oxide-wrapped gold nanorods as robust nanoplatform for ultrafast near-infrared SERS bioimaging. Int J Nanomed.

[53] Turcheniuk K, Dumych T, Bilyy R, Turcheniuk V, Bouckaert J, Vovk V (2016). Plasmonic photothermal cancer therapy with gold nanorods/reduced graphene oxide core/shell nanocomposites. RSC Adv.

[54] Feng L, Liu Z (2011). Graphene in biomedicine: opportunities and challenges. Nanomedicine.

[55] Shareena TPD, McShan D, Dasmahapatra AK, Tchounwou PB (2018). A review on graphene-based nanomaterials in biomedical applications and risks in environment and health. Nano-Micro lett.

[56] Wang S, Zhao X, Wang S, Qian J, He S (2016). Biologically inspired polydopamine capped gold nanorods for drug delivery and light-mediated cancer therapy. ACS Appl Mater Interfaces.

[57] Wu D, Chen X, Zhou J, Chen Y, Wan T, Wang Y (2020). A synergistic optical strategy for enhanced deep-tumor penetration and therapy in the second near-infrared window. Mater Horiz.

[58] Encinas-Basurto D, Ibarra J, Juarez J, Pardo A, Barbosa S, Taboada P (2018). Hybrid folic acid-conjugated gold nanorods-loaded human serum albumin nanoparticles for simultaneous photothermal and chemotherapeutic therapy. Mater Sci Eng C Mater Biol Appl.

[59] Tebbe M, Kuttner C, Männel M, Fery A, Chanana M (2015). Colloidally stable and surfactant-free protein-coated gold nanorods in biological media. ACS Appl Mater Interfaces.

[60] Sahin Z, Demir YK, Kayser V (2016). Global kinetic analysis of seeded BSA aggregation. Eur J Pharm Sci.

[61] Borzova VA, Markossian KA, Chebotareva NA, Kleymenov SY, Poliansky NB, Muranov KO (2016). Kinetics of thermal denaturation and aggregation of bovine serum albumin. PLoS One.

[62] Wang S-L, Lin S-Y, Li M-J, Wei Y-S, Hsieh T-F (2005). Temperature effect on the structural stability, similarity, and reversibility of human serum albumin in different states. Biophys Chem.

[63] Liu J, Ma W, Kou W, Shang L, Huang R, Zhao J (2019). Poly-amino acids coated gold nanorod and doxorubicin for synergistic photodynamic therapy and chemotherapy in ovarian cancer cells. Biosci Rep.

[64] Craciun A, Focsan M, Gaina L, Astilean S (2017). Enhanced one-and two-photon excited fluorescence of cationic (phenothiazinyl) vinyl-pyridinium chromophore attached to polyelectrolyte-coated gold nanorods. Dyes Pigm.

